# CARK1 phosphorylates subfamily III members of ABA receptors

**DOI:** 10.1093/jxb/ery374

**Published:** 2018-10-30

**Authors:** Xiaoyi Li, Xiangge Kong, Qi Huang, Qian Zhang, Hu Ge, Liang Zhang, Gaoming Li, Lu Peng, Zhibin Liu, Jianmei Wang, Xufeng Li, Yi Yang

**Affiliations:** Key Laboratory of Bio-Resources and Eco-Environment of Ministry of Education, College of Life Sciences, Sichuan University, Chengdu, China

**Keywords:** ABA signaling, abscisic acid, abiotic stress, *Arabidopsis thaliana*, drought, phosphorylation, seed germination, transient luciferase assay

## Abstract

Abscisic acid (ABA) plays a vital role in responses to abiotic stresses that allow plants to cope with environmental challenges. In this study, we analyzed ABA receptors of subfamily III as the potential targets of Cytosolic ABA Receptor Kinase 1 (CARK1). We previously found that CARK1 phosphorylated the subfamily III member RCAR11 at a distinct threonine residue (T78). Our study now shows the physical interaction of CARK1 with the receptors RCAR12/13/14 *in vitro* and *in vivo*. The catalytically inactive form CARK1-N204A did not interact with the receptors. Phosphorylation of these ABA receptors *in vitro* occurred at a serine/threonine amino acid residue corresponding to T78 in RCAR11, which is located in the loop of β3 within a conserved site. Further analysis revealed that the phosphorylation of RCAR11^T78^ could increase the sensitivity of the *pyr1pyl1pyl2pyl4* quadruple mutant (1124) to ABA, including the inhibition of root elongation and increasing drought tolerance. The analysis of *CARK1*:1124 complementation and the expression of ABA-related genes indicated that CARK1 could rescue the insensitivity of 1124 to ABA. Our results indicate that CARK1 tends to phosphorylate subfamily III ABA receptors, and the phosphosites RCAR11^T78^, RCAR12^T105^, RCAR13^T101^, and RCAR14^S81^ are the major sites involved in the activation of the ABA response pathway.

## Teaser

CARK1 preferentially interacts with and phosphorylates ABA receptors of subfamily III; the phosphorylation site RCAR11^T78^ plays a substantial role in the activation of the ABA response pathway.

## Introduction

The phytohormone abscisic acid (ABA) plays an important role in plant growth and developmental processes as well as in plant responses to environmental stresses, including salinity and drought (Finkelstein *et al.*, 2002; Yoshida *et al.*, 2002; Cutler *et al.*, 2010; Fujita *et al.*, 2011). Core ABA signaling components found in Arabidopsis are conserved, such as ABA receptors (Hauser *et al.*, 2011). When binding to ABA, pyrabactin resistance 1 (PYR1)/PYR1-like (PYL)/regulatory components of ABA receptors (RCAR) (hereafter referred to as RCARs) interact with and inhibit clade A protein phosphatase 2C (PP2C). This releases SNF1-related protein kinase 2 (SnRK2)-type protein kinases from inhibition, subsequently allowing SnRK2 to phosphorylate and activate downstream components, such as transcription factors and ion channels (Furihata *et al.*, 2006; Fujita *et al.*, 2009; Lee *et al.*, 2009; Zhou *et al.*, 2015).

The Arabidopsis genome encodes 14 RCARs, which are divided into three subfamilies according to their sequence homology, namely subfamily I members (RCAR1–RCAR4), subfamily II (RCAR5–RCAR10) and subfamily III members (RCAR11–RCAR14) (Ma *et al.*, 2009; Park *et al.*, 2009; Nishimura *et al.*, 2010). All of them show overlapping patterns of gene expressions (Winter *et al.*, 2007; Gonzalez-Guzman *et al.*, 2012; Antoni *et al.*, 2013), and functional redundancy among them can be expected. For instance, RCAR11 and RCAR14 are found to be ubiquitylated by RSL1 and degraded by the 26S proteasome in *Arabidopsis thaliana* (Eduardo *et al.*, 2015). At basal ABA levels, subfamily I members efficiently regulated ABA signaling, whereas those of subfamily II are moderately inhibited by PP2C activities (Zhao *et al.*, 2013; Tischer *et al.*, 2017). From a biochemical point of view, the receptors of subfamily I and II are characterized by a monomeric state, while those in subfamily III are in a dimeric state (Nishimura *et al.*, 2009; Dupeux *et al.*, 2011; Hao *et al.*, 2011). The dimeric receptors show a lower affinity for ABA than the monomeric receptors (Ma *et al.*, 2009; Santiago *et al.*, 2009). Previous studies also found that subfamily III receptors exhibited ABA-dependent inhibition of PP2Cs, whereas other members inhibited the activities of PP2Cs even in the absence of ABA (Fujii *et al.*, 2009; Miyazono *et al.*, 2009; Tischer *et al.*, 2017). The phenotypic analysis of ABA receptor mutants revealed that single mutants exhibited normal ABA sensitivity, except for *pyl8*, the lateral root growth of which was more sensitive to inhibition by ABA (Zhao *et al.*, 2014). The quadruple mutant *pyr1pyl1pyl2pyl4* (abbreviated as 1124) which is deficient in the three dimeric receptors (RCAR11, RCAR12, and RCAR14) and a monomeric receptor (RCAR10) shows a strong ABA insensitivity, including ABA-mediated inhibition of germination, root growth, and ABA-induced stomatal closure, providing evidence for the importance of the dimeric receptors in Arabidopsis (Park *et al.*, 2009; Nishimura *et al.*, 2010; Gonzalez-Guzman *et al.*, 2012). However, the quadruple mutant still responds to ABA to some extent, indicating that all of the ABA receptors function similarly in ABA signaling (Gonzalez-Guzman *et al.*, 2012).

The diversity of responses regulated by ABA and its dynamics in stress development associated with different ABA levels might require a large number of different ABA receptors to adjust signal transduction and metabolism in plants. Phosphorylation, a type of post-translational modification, has been found to play an important role in ABA signaling (Yoshida *et al.*, 2002; Fujita *et al.*, 2009; Zhou *et al.*, 2015). The phosphorylation of RCAR11 or RCAR12 by target of rapamycin 1 (TOR1) would suppress ABA binding, and EL1-like kinases phosphorylate RCAR12 and then promote its degradation by 26S protease (Chen *et al.*, 2018; Wang *et al.*, 2018), suggesting that these kinases are negatively involved in ABA signaling. A recent study elaborates that the phosphorylation of RCAR3 and RCAR11 by CARK1 (Cytosolic ABA Receptor Kinase 1) positively promotes ABA signaling, and the N204 of CARK1, the critical active site, accounts for the interactions between CARK1 and RCAR3/RCAR11 (Zhang *et al.*, 2018). CARK1 belongs to a putative Ser/Thr protein kinase RLCK VIII subfamily in Arabidopsis. Here, we report that CARK1 interacts with and phosphorylates RCAR12/13/14, and find the major phosphosites T105 of RCAR12, T101 of RCAR13, and S81 of RCAR14. Moreover, we reveal that a major phosphorylation site (T^78^) in RCAR11 is closely linked to the regulation of ABA-induced germination, root length, and drought tolerance, and affects the expression of ABA-related genes.

## Materials and methods

### Plant materials

Arabidopsis plants used in this study were in the Columbia (Col-0, WT), 1124 quadruple mutant (Gonzalez-Guzman *et al.*, 2012), or Landsberg erecta ecotype (*Ler*) background. The *CARK1* T-DNA insertion mutant *cark1* (SALK_113377) was obtained from the Arabidopsis Biological Resources Center. Plants were grown in soil–vermiculite mixtures at 22 °C under 60% relative humidity with a photoperiod of 16 h light/8 h dark and 120 μmol m^–2^ s^–1^. For plate culture, seeds were first soaked in distilled water for 3 d at 4 °C. After stratification, seeds were surface sterilized and germinated on solid Murashige and Skoog (MS) medium containing 2% sucrose and 0.8% agar, pH 5.8.

### *In vitro* GST pull-down assay

*RCAR* genes were cloned into the pGEX-6p-1 expression vector (Novagen, WI, USA) with a glutathione *S*-transferase (GST) tag fused at the N-terminal end. The primers are listed in Supplementary Table S1 at *JXB* online. These plasmids were transfected into *Escherichia coli* strain Rosetta (DE3), and protein expression and purification were performed for CARK1-KD (CARK1 kinase domain, residues 50–353) as previously described (Zhang *et al.*, 2018). GST–RCARs were added to 25 μl of glutathione *S*–Sepharose 4B (GS4B, GE Healthcare, PA, USA) resin. His-CARK1-KD^N204A^ or His-CARK1-KD was co-incubated with the GST–RCAR-bound resin for 1 h at room temperature. The resin was then extensively rinsed with buffer to remove unbound proteins. The resins were finally resuspended in 100 μl of buffer, and 10 μl of the suspension was subjected to 10% SDS–PAGE for analysis. A 0.2 μg aliquot of inputs was used in the assay, and the anti-His antibody (mouse derived Mab, TransGen Biotech; Beijing, China) was used in the western blot analysis.

### Bimolecular fluorescence complementation (BiFC) assay

The full-length coding sequence (CDS) of *RCAR9/12/13/14* was individually cloned into the binary nYFP (yellow fluorescent protein) vector via *Bam*HI and *Sal*I sites to generate the RCAR9/RCAR12/13/14–YFP^N^ fusion protein. YFP^C^–CARK1 and YFP^C^–CARK1^N204A^ constructions were as described by Zhang *et al.* (2018). Primer pairs for the construction of the vectors are listed in Supplementary Table S1. The BiFC assay was performed as previously described (Song *et al.*, 2011). Briefly, various nYFP and cYFP fusion vectors were introduced into *Agrobacterium tumefaciens* strain GV3101. After incubation, *Agrobacterium* cells were harvested and resuspended in infiltration buffer (0.2 mM acetosyringone, 10 mM MgCl_2_, and 10 mM MES) to identical concentrations (OD_600_=0.5), and then transferred into the leaf cells of *Nicotiana benthamiana* by a needleless syringe. After 2 d, cells with YFP fluorescence were observed and imaged with a confocal laser-scanning microscope. The experiment was repeated three times, each time with three or four biological replicates.

### Co-immunoprecipitation (Co-IP) assay

The full-length CDS of *RCAR12/13/14* was cloned into the pHB vector via *Bam*HI and *Sal*I sites to generate pHB-3*flag-*RCAR12/13/14*, respectively. The full-length CDS of *CARK1* and *CARK1*^*N204A*^ were cloned into the pHB vector via *Sal*I and *Xba*I sites to generate pHB-3*HA-*CARK1* and pHB-3*HA-*CARK1*^*N204A*^. The recombined plasmids were transiently expressed in *N. benthamiana* by *Agrobacterium* infiltration as described above. Proteins were extracted and resuspended in IP buffer [50 mM HEPES, pH 7.5, 150 mM NaCl, 1% polyvinyloly pyrrolidone (PVPP), 10% glycerol, 1% Triton X-100, 2 mM DTT, 2 mM phenylmethylsulfonate fluoride (PMSF), and 1× protease inhibitor cocktail (Roche, Basel, Switzerland)]. After extraction in IP buffer, crude protein extracts (Input) were used for immunoprecipitation with Anti-Flag^**®**^ M2 Magnetic Beads (Sigma-Aldrich). The beads were resuspended in 2× sample loading buffer and boiled at 98 °C for 10 min. The supernatant of the crude extracts was used as the input. Anti-HA and anti-Flag antibodies (Bioworld, Minneapolis, USA) were used in the immunoblot. Co-IP) was performed as previously described (Fiil *et al.*, 2008).

### *In vitro* kinase assay

*RCAR12*, *RCAR12*^*T105A*^, *RCAR13*, *RCAR13*^*T101A*^, *RCAR14*, and *RCAR14*^S81A^ were cloned into the pET28a expression vector with a 6× His tag fused at the C-terminal side. The plasmids were then transformed into *E. coli* strain Rosetta (DE3). After the OD_600_ reached 0.5, the culture was cooled to 16 °C and supplemented with 0.5 mM isopropyl-β-d-thiogalactopyranoside (IPTG). The cells were harvested by centrifugation and the pellets were resuspended in lysis buffer containing 20 mM Tris–HCl (pH 8.0), 150 mM NaCl, and 5 mM MgCl_2_. The fusion proteins were purified by Ni-NTA affinity chromatography (Thermo Fisher Scientific, Rockford, IL, USA).

For autophosphorylation and transphosphorylation assays, 1 μM CARK1-KD or CARK1-KD mutant was diluted to 25 μl using reaction buffer (20 mM Tris, pH 7.5, 100 mM NaCl, 10 mM MgCl_2_, 2 mM DTT, and 10 mM ATP). The reaction mixture was incubated at 30 °C for 1 h and terminated by adding an equal volume of 2× SDS loading buffer. Horseradish peroxidase (HRP)-conjugated Phosphor-Threonine Antibody (Cell Signaling Technology, Beverly, MA, USA) was used. Western blots were developed with the ECL chemilluminesence detection system (Bio-Rad, Hercules, CA, USA).

### Genotyping analysis of the CARK1 mutant and generation of various CARK1 transgenic plants

The construct for the complemented lines of Flag-tagged *RCAR11/RCAR11*^*T78A/E*^ or HA-tagged *CARK1* in the 1124 background (abbreviated as R11/R11A/R11E:1124) were generated as previously described (Zhang *et al.*, 2018). The resulting constructs were transformed into *N. benthamiana* strain GV3101, which was subsequently infiltrated into the WT plants using the ‘floral dip’ method (Clough and Bent, 1998). All transgenic plants were screened on MS medium supplemented with hygromycin, and mRNA levels were verified with real-time PCR (RT-PCR) assays.

### Physiological analysis

The germination assay was carried out as described by Lee *et al.* (2015) and the root length assay was as described by Cui *et al.* (2012). Approximately 100 seeds were placed on solid MS agar medium with different concentrations of ABA. Germination rates were scored 4 d after incubation. For root growth assay, >50 seeds from each line were first germinated vertically on MS medium for 3 d. Then, 20 seedlings of each line with similar root lengths were transferred in a vertical position to 1/2 MS medium supplemented with or without 40 μM or 30 μM ABA. The root length was determined 1 week after transfer. For the drought tolerance test, 2-week-old seedlings grown under the same conditions were subjected to drought stress treatment by withholding water for 10 d. After rehydration for 2 d, the morphological changes of plants were recorded.

### Statistical analysis

Data are represented as the means ±SD. Statistical analysis was performed using Student’s *t*-test. Values of *P*<0.05 were considered significant, and values of *P*<0.01 were considered more significant.

### RNA isolation and qRT-PCR analysis

Two-week-old seedlings were incubated in liquid MS medium with or without 50 μM ABA for 3 h. Total RNAs was extracted using the RNAiso Plus reagent from Takara (Otsu, Japan). The cDNAs were synthesized from 1 μg of total RNAs using the PrimeScript RT-PCR reagent Kit of Takara. qRT-PCR was performed using the Bio-Rad CFX96 real-time PCR detection system and SYBR Premix Ex Taq II from Takara. ACTIN2/8 was used as an internal control. RT-PCR was conducted with the gene-specific primers described by Zhang *et al.* (2018).

### Transient luciferase (LUC) assay

Transcription activity of the interaction between CARK1/CARK1^N204A^ (CARK1m) and RCARs against promoters of ABA signaling-responsive marker genes was determined using the dual luciferase assay of transiently transformed Arabidopsis protoplasts. Marker gene promoters (*RAB18* and *RD29B*) were subcloned into the transient expression reporter vector pGreenII0800-LUC that contained the *Cauliflower mosaic virus* (CaMV) 35S promoter–REN cassette and the promoterless LUC cassette. *RCAR12/13/14* were subcloned into the vector pBI221 via *Xba*I and *Bam*HI sites. The internal control construct p35S::*GUS* and pBI221-*CARK1/CARK1m* vectors were used as effector constructs as previously described (Zhang *et al.*, 2018). Primers used in this study are listed in Supplementary Table S1.

Preparation of Arabidopsis protoplasts of the *cark1* T-DNA mutant plants and subsequent transfection of protoplasts were performed as described (Yoo *et al.*, 2007). A no-effector construct was used as control. When indicated, 10 μM ABA was added to the incubation buffer immediately after transfection. Luciferase activity was measured by an LMax II^384^ luminometer (Molecular Devices, Bad Wildbad, Germany) using the Dual-Luciferase Reporter Assay System from Promega (Madison, WI, USA). Data are reported as the LUC/REN ratio. Three independent experiments were performed, and for each sample three technical replicates were analyzed.

## Results

### CARK1 interacts directly with RCAR12, RCAR13, and RCAR14

**Fig. 1.**
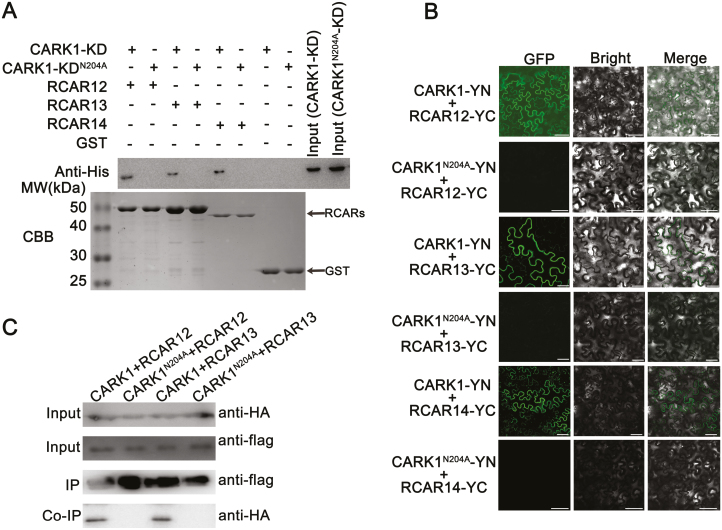
CARK1 interacts with RCAR12, RCAR13, and RCAR14 *in vitro* and *in vivo.* (A) *In vitro* GST pull-down assay of CARK1-KD and CARK1-KD^N204A^ interaction with RCAR12, RCAR13, or RCAR14. A 50 μg aliquot of 6× His-fused CARK1-KD or CARK1-KD^N204A^ was incubated with 50 μg of GST-tagged RCAR12, RCAR13, or RCAR14. The immunoprecipitated proteins were analyzed by SDS–PAGE (bottom) and immunoblotting with antibodies against the His-tag (top). A 0.1 μg aliquot of both input proteins CARK1-KD and CARK1-KD^N204A^ was used. (B) BiFC assay validates the interactions of RCAR12, RCAR13, and RCAR14 with CARK1, but not with mutant CARK1^N204A^, in the leaves of *N. benthamiana*. nYFP was fused to the full-length CARK1 and mutant CARK1^N204A^ to form CARK1–nYFP and CARK1^N204A^–nYFP, and cYFP was fused to RCAR12, RCAR13, and RCAR14 to form cYFP–RCAR12, cYFP–RCAR13, and cYFP–RCAR14, respectively. Pictures were taken using a confocal laser-scanning microscope (LSM 710, Carl Zeiss). Scale bars=50 µm. (C) Co-immunoprecipitation (Co-IP) experiment of 3*HA-tagged CARK1/CARK1^N204A^ and 3*Flag-tagged RCAR12 or RCAR13 was performed in *N. benthamiana*. Protein extracts were immunoprecipitated using an anti-Flag antibody and subjected to immunoblot analysis with an anti-HA antibody. All the experiments were conducted with at least three biological replicates.

In a previous study, the results from the yeast two-hybrid assay demonstrated that CARK1 interacted with RCARs *in vitro* (Zhang *et al.*, 2018). Here, a GST pull-down assay revealed that CARK1-KD interacted with RCAR12, RCAR13, and RCAR14 (Fig. 1A). However, these interactions were completely impaired by a single amino acid substitution in the kinase activity center of CARK1 (CARK1-KD^N204A^) (Fig. 1A). The BiFC assay showed that co-expression of CARK1–nYFP/cYFP–RCAR12, CARK1–nYFP/cYFP–RCAR13, or CARK1–nYFP/cYFP–RCAR14 in the leaves of *N. benthamiana* yielded YFP signals in the cytosol (Fig. 1B), while co-expression of a series of negative controls did not generate fluorescence (Supplementary Fig. S1A). The result of western blot showed that CARK1^N204A^ was ectopically expressed in *N. benthamiana* (Supplementary Fig. S1B). However, no YFP signal was detected in leaves after co-expression of CARK1^N204A^–nYFP/cYFP–RCAR12, CARK1^N204A^–nYFP/cYFP–RCAR13, or CARK1^N204A^–nYFP/cYFP–RCAR14 (Fig. 1B). Similarly, the Co-IP assay revealed that the full-length CARK1, but not CARK1^N204A^, could interact with RCAR12 and RCAR13 in *N. benthamiana* (Fig. 1C). The previous study showed that CARK1 could interact with the subfamily I member, RCAR3; therefore, we also tested the interaction between CARK1 and RCAR9 (belonging to subfamily II of ABA receptors) (Zhang *et al.*, 2018). The result showed that there was no fluorescence when CARK1 and RCAR9 were co-expressed (Supplementary Fig. S1A). Our investigations confirm that CARK1 indeed physically interacts with RCAR12, RCAR13, or RCAR14 in plant cells and the N204 of CARK1 is critical for CARK1’s kinase activity and for its interactions with RCAR12, RCAR13, and RCAR14.

### CARK1 phosphorylates RCAR12/13/14 *in vitro*

**Fig. 2.**
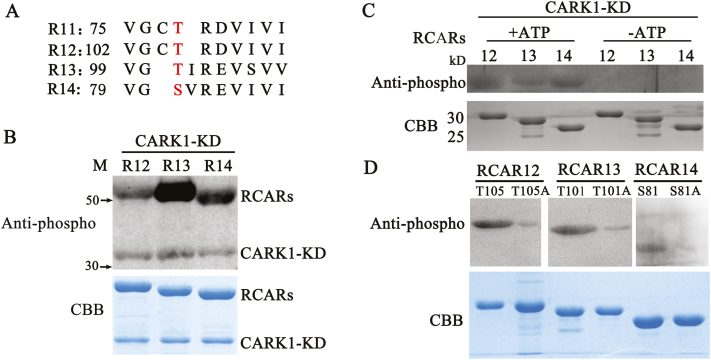
CARK1 phosphorylates RCAR12, RCAR13, and RCAR14. (A) Alignment of the phosphorylation sites with subfamily III receptor RCARs (R11–R14). (B–D) *In vitro* kinase assay. Phosphorylation of GST–RCAR12/13/14 (abbreviated as R12/13/14) by CARK1-KD (B). Phosphorylation of His-RCAR12/13/14 by CARK1-KD (C). Determination of the potential phosphorylation site of RCAR12/13/14 (D). Anti-phosphothreoinine antibody was used for the analysis of the western blot. The loading amounts of proteins were visualized on the Coomassie Brilliant Blue- (CBB) stained gel (bottom panel). Experiments were repeated at least three times.

Based on the finding that CARK1 interacted with RCAR12/13/14, we next tested whether they could be phosphorylated by CARK1 *in vitro*. Alignment analysis revealed that ABA receptors are highly conserved (Fig. 2A) (Yin *et al.*, 2009), suggesting their possible phosphorylation by CARK1. Therefore, we examined the ability of CARK1 to phosphorylate RCAR12/13/14 using a kinase assay *in vitro*. CARK1-KD was cloned and expressed with a His-tag, and RCARs as the substrates were purified as GST-tag proteins in an *E. coli* system. After separating the protein by SDS–PAGE, we detected the phosphorylated proteins on the gel using an anti-phosphothreonine antibody which could recognize the Ser and Thr phosphorylation sites and visualized total proteins with Coomassie Brilliant Blue (CBB) staining. As shown in Fig. 2B, an autophosphorylation band of ~38 kDa corresponding to His-CARK1-KD was observed in all samples, and the band of ~55 kDa showed that RCAR12, RCAR13, and RCAR14 were phosphorylated by CARK1. Similarly, CARK1 could also phosphorylate His-RCAR12, RCAR13, and RCAR14 (Fig. 2C). However, RCAR12, RCAR13, and RCAR14 were not phosphorylated by CARK1-KD without ATP (Fig. 2C). In a previous study, CARK1 was shown to phosphorylate the T^78^ site of RCAR11 *in vitro* and *in vivo* (Zhang *et al.*, 2018). The T^78^ site of RCAR11 is located in the loop of β3 (Santiago *et al.*, 2009). Protein kinases usually phosphorylated Ser and Thr residues on S/T-P motifs; the possible phosphorylation sites can be predicted in RCAR12/13/14 by an analysis of their homologous sequences (Fig. 2A), namely on RCAR12^T105^, RCAR13^T101^, and RCAR14^S81^. The analysis of the kinase assay *in vitro* showed that mutations of these sites to alanine (preventing phosphorylation) resulted in significantly suppressed phosphorylation by CARK1 (Fig. 2D). Therefore, our results further confirm the roles of these sites in phosphorylation (T78 of RCAR11, T105 of RCAR12, T101 of RCAR13, or S81 of RCAR14) and indicate a conserved CARK1-mediated phosphorylation of RCARs.

### The phosphorylation of RCAR11 affects the responses of plants to ABA

**Fig. 3.**
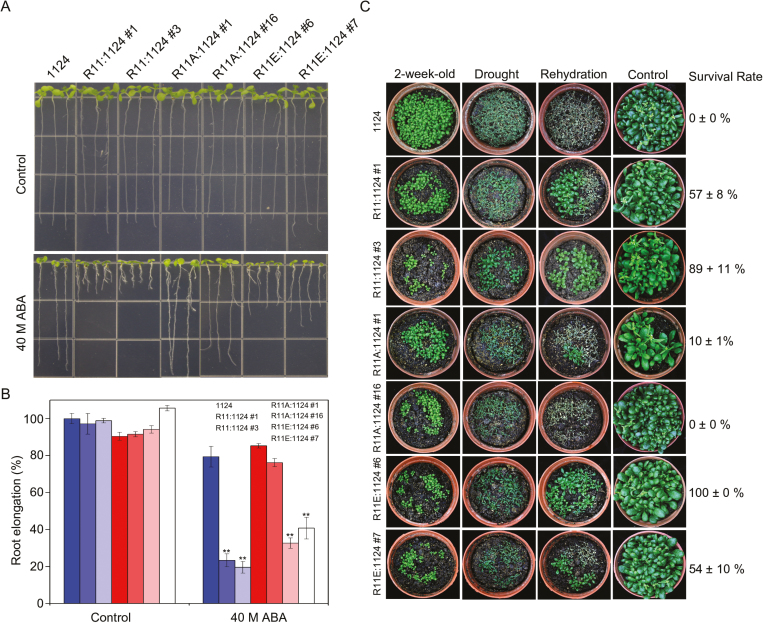
Phosphorylation state of RCAR11 affects the response of plants to ABA. (A) The root architecture of plant seedlings was documented at 7 d post-transfer to medium (1/2 MS, 1% sucrose) with or without ABA. Seedlings were 3 d old at the time of transfer and had equal root lengths at that time. (B) Statistical analysis of the ABA-inhibited root growth in (A). Data are the means ±SD (*n*=20). (C) Drought tolerance assay. Two-week old plants before drought (top), after drought (second line), 2 d after rehydration (third line), and control without drought stress (bottom line). 1124, ABA receptor *pyr1pyl1pyl2pyl4* quadruple mutant; R11:1124, RCAR11 overexpression in the 1124 quadruple mutant plants; R11A:1124, the phosphor-defective forms of RCAR11^T78A^ overexpressed in the 1124 quadruple mutant plants; R11E:1124, a phosphor-mimic form of RCAR11^T78E^ overexpressed in the 1124 quadruple mutant plants. *P<0.05, **P<0.01, Student’s *t*-test. All physiological analyses were conducted in triplicate.

ABA is a key factor in controlling seedling growth including root length (Park *et al.*, 2009; Nishimura *et al.*, 2010; Gonzalez-Guzman *et al.*, 2012). To elucidate whether the phosphorylation of ABA receptors by CARK1 has a corresponding function in ABA signaling, we generated various RCAR11 (R11) complemented transgenic lines in the 1124 mutant including the WT (R11:1124), phosphor-defective and phosphor-mimic forms on T78 (R11A:1124 and R11E:1124), respectively. The analysis of root elongation revealed that R11:1124 and R11E:1124 had shorter roots than those of 1124 in the presence of ABA, while the relative root length of 1124 was similar to that of R11A:1124 (Fig. 3A, B). However, the root length of the plants exhibited no difference in the absence of ABA (Fig. 3A, B). These results indicate that the phosphorylation of RCAR11 is a positive regulator of root growth inhibition in response to ABA.

To confirm our observations further, we next exposed the 1124 and the complemented plants to a dehydration condition by withholding water. There was no significant difference among them when plants grew under well-watered conditions, but all of them displayed wilting phenotypes after drought treatment for 10 d (Fig. 3C). However, after rewatering for 2 d, >50% of R11:1124 and R11E:1124 transgenic plants recovered, while R11A:1124 and 1124 plants did not recover, with the exception that R11A:1124 #1 was ~10% recovered (Fig. 3C). These results indicated that transgenic plants bearing phosphor-defective forms of RCAR11 (R11A:1124) almost completely lost ABA sensitivity and drought tolerance compared with the WT form of RCAR11 (R11:1124), while the phosphor-mimic form of RCAR11 (R11E:1124) could notably complement the phenotypes (Fig. 3).

### CARK1 rescues the sensitivity of 1124 quadruple mutant plants to ABA

**Fig. 4.**
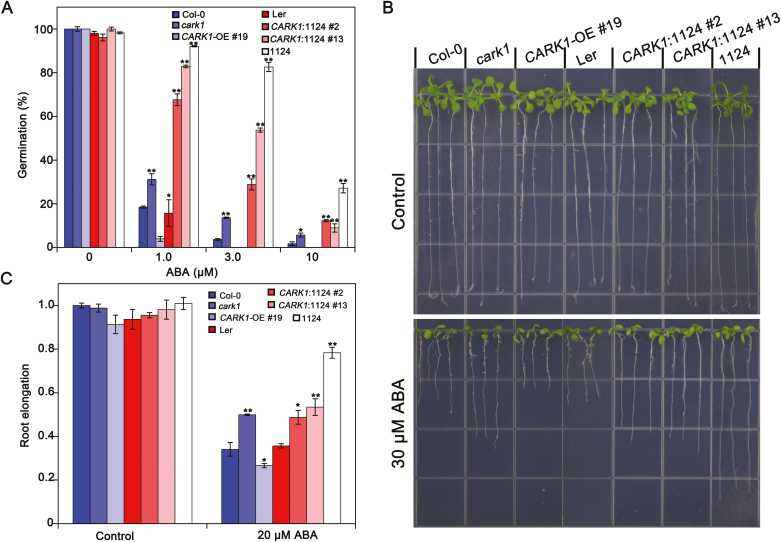
RCARs act downstream of CARK1 in the ABA-induced inhibition of seed germination and root length. (A) Seed germination assay. Seeds (>100) were scored 3 d after stratification on MS medium supplemented with different concentrations of ABA. Values are the means ±SD (*n*=3). (B) The root architecture of seedlings was documented at 7 d post-transfer to media (1/2 MS, 1% sucrose) with or without ABA. Seedlings were 3 d old at the time of transfer and had equal root lengths at that time. (C) Statistical analysis of ABA-inhibited root growth in (B). Data are the means ±SD (*n*=20). Col-0, Columbia ecotype; *cark1*, CARK1 T-DNA mutant; *CARK1*-OE 19#, plants overexpressing *CARK1* in Col-0; *Ler* (Landsberg erecta ecotype); *CARK1*:1124, overexpression of *CARK1* in the 1124 quadruple mutant plants; 1124, ABA receptor *pyr1pyl1pyl2pyl4* quadruple mutant. *P<0.05, **P<0.01, Student’s *t*-test. All physiological analyses were conducted in triplicate.

Subsequently, a complementation test was conducted. *CARK1* was ectopically expressed in the 1124 mutant. Expression of the *CARK1* transgene was detected in *CARK1*:1124 (lines #2 and #13) T_3_ complementation lines by RT-PCR and protein levels (Supplementary Fig. S2). These complementation progeny were used in the phenotypic analysis of ABA responses to determine whether the mutant phenotypes were rescued. As described previously (Park *et al.*, 2009; Gonzalez-Guzman *et al.*, 2012; Zhang *et al.*, 2018), *cark1* and1124 mutants were insensitive to ABA compared with WT plants during the germination and post-germination stages. The germination of *CARK1*:1124 transgenic seeds was higher than that of *CARK1*-OE #19 but lower than that of 1124 under ABA treatment (Fig. 4A). As shown in Fig. 4B and C, compared with the WT, *CARK1*-OE #19 showed increased sensitivity to ABA-inhibited primary root growth. The insensitivity of 1124 to ABA-mediated inhibition of root growth was notably diminished when *CARK1* was overexpressed in 1124 (*CARK1*:1124) (Fig. 4B, C). These results indicate that the ABA-sensitive phenotypes of *CARK1*:1124 were partly rescued by reciprocal overexpression of *CARK1*.

**Fig. 5.**
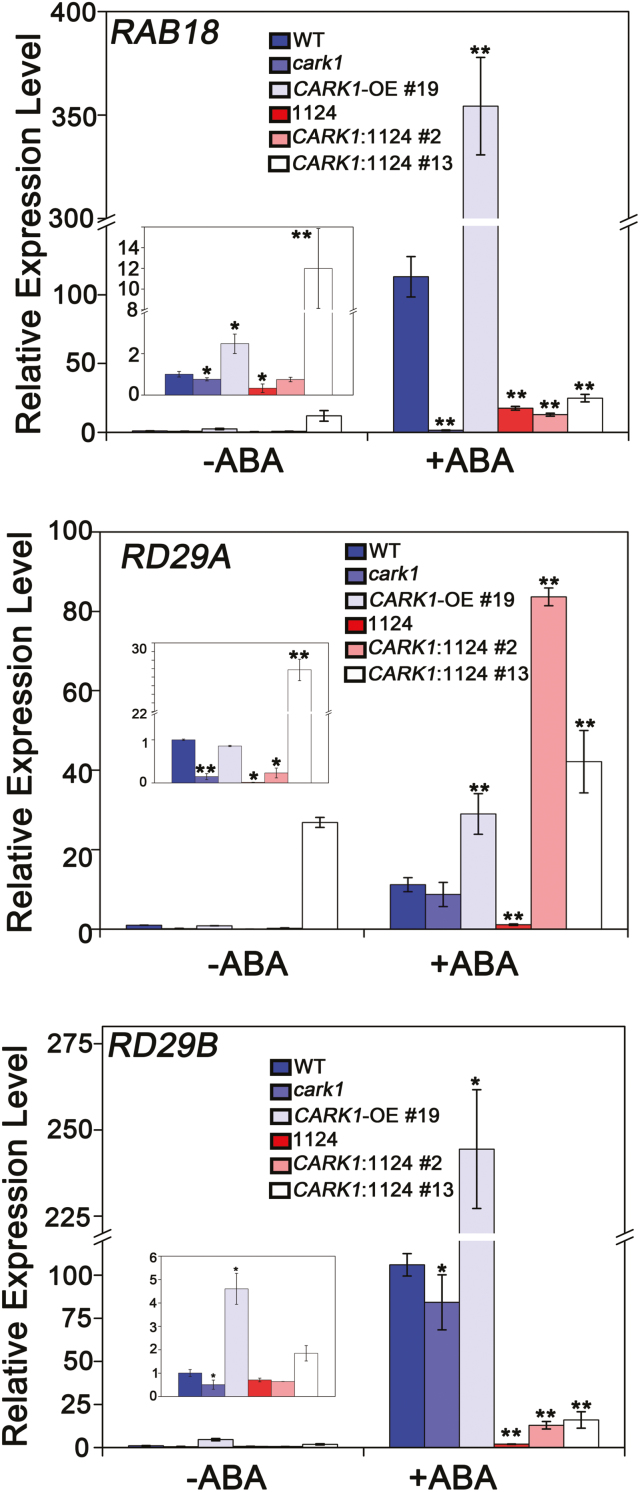
CARK1 attenuates the expression of several ABA-responsive genes. Relative expression levels of the ABA-responsive genes *RAB18* (A), *RD29A* (B), and *RD29B* (C) in plants. Two-week-old seedlings were incubated in MS liquid medium with or without 50 μM ABA for 3 h. The transcriptional levels were determined by qRT-PCR analysis. Values are the means ±SD (*n*=3). *ACTIN2/8* was used as an internal control. WT, Columbia ecotype; *cark1*, CARK1 T-DNA mutant; *CARK1*-OE 19#, plants overexpressing *CARK1* in Col-0; 1124, ABA receptor *pyr1pyl1pyl2pyl4* quadruple mutant; *CARK1*:1124, overexpression of *CARK1* in the 1124 quadruple mutant plants.*P<0.05, **P<0.01, Student’s *t*-test. The experiment was conducted by triplicate.

Furthermore, we examined the mechanism whereby CARK1 mediates the ABA signaling pathway in reciprocal transgenic plants. The expression of several ABA-related genes (*RAB18*, *RD29A*, and *RD29B*) was measured by qRT-PCR. The results showed that the expression levels of *RAB18*, *RD29A*, and *RD29B* in *CARK1*:1124 #13 were higher than those in 1124 without ABA treatment (Fig. 5). After the treatment with exogenous ABA for 3 h, the expression of these ABA-related genes in *CARK1*:1124 (lines #2 and #13) was significantly increased and induced compared with that in the WT. Taken together, the transgenic plants, overexpressing *CARK1* in the 1124 background, promoted the expressions of ABA-related genes after treatment with exogenous ABA.

From these results, we further revealed the significance of phosphorylation of ABA receptors in fine-tuning ABA signaling. On the other hand, CARK1 promotes ABA-mediated germination, root growth, and gene expression. Collectively, these studies support the hypothesis that kinase activity is critical for the function of CARK1 in ABA signaling.

### CARK1 and RCARs synergistically promotes ABA-responsive gene expression in Arabidopsis protoplasts.

**Fig. 6.**
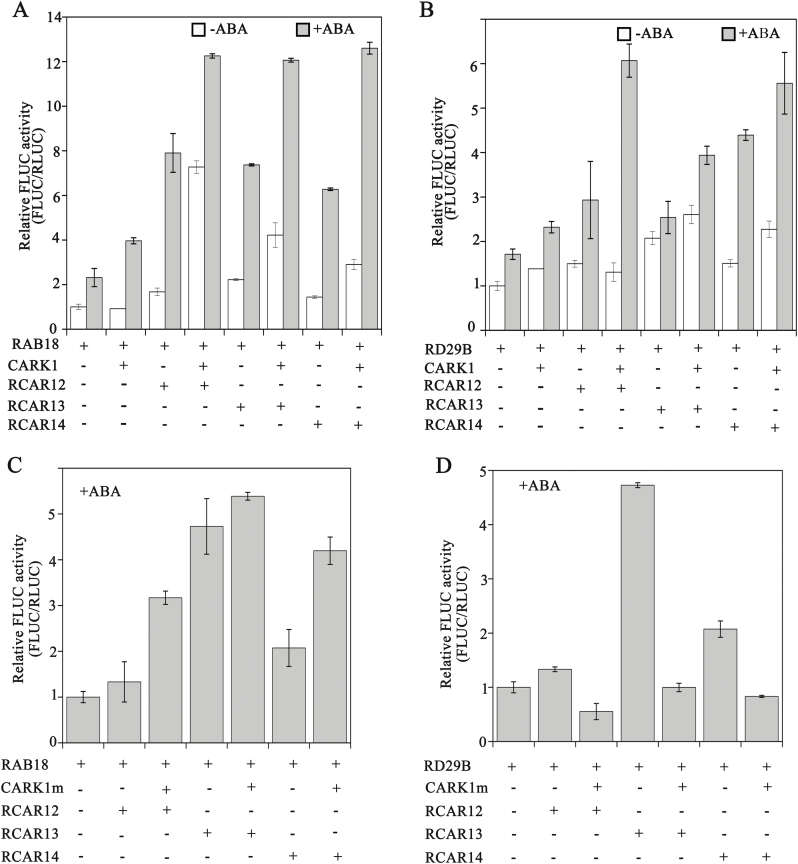
CARK1 and subfamily III ABA receptors synergistically regulate the expression of the reporter gene driven by the *RAB18* or *RAB29B* promoter. Protoplasts of *cark1* were transfected with 10 μg of effector DNA *CARK1* (A and B) or *CARK1*^*N204A*^ (abbreviated as CARK1m; C and D) for expression of the different RCARs (RCAR12/13/14) in the absence and presence of 10 μM exogenous ABA. As a negative control, empty effector vectors were used, taking the place of the effector plasmid. The relative activity of the vector control was set at 1. Experiments were repeated at least three times. Vertical bars indicate ±SD of three replicates.

Transient LUC assays in *cark1* mutant protoplasts were used to determine the effect of the interaction of CARK1 with ABA receptors on the expressions of the LUC gene driven by the *RAB18* or *RD29B* promoter (Fig. 6). In the absence of exogenous ABA, the single transfection of CARK1 or RCAR12/13/14 had a minimal effect on the expression of *RAB18-FLuc* (1.2- to 2.3 fold) (Fig. 6A). On the other hand, the relative luciferase activity (LUC/REN) showed a significant enhancement (2.5- to 8-fold) when CARK1 and RCARs were co-transfected in *cark1* protoplasts (Fig. 6A). Co-expression of CARK1 and RCARs in the presence of ABA noticeably increased the ABA response, on average >11-fold for the expression of *RAB18-FLuc* (Fig. 6A).

Similarly, in the absence of exogenous ABA, the single transfection of CARK1 or RCAR13/14 had a minimal effect on the expression of *RD29B-FLuc* (2.5- to 4-fold) (Fig. 6B). On the other hand, the relative luciferase activity (LUC/REN) showed a significant enhancement (4- to 6-fold) when CARK1 and RCARs were co-transfected in *cark1* protoplasts in the presence of ABA (Fig. 6B).

However, there were no obviously increased activities of luciferase when CARK1m and RCAR12/13/14 were co-expressed in *cark1* protoplasts (Fig. 6C, D). Therefore, these findings support the notion that the phosphorylation of RCAR12/13/14 by CARK1 induces the expression of ABA-responsive genes and synergistically regulates ABA signaling.

## Discussion

Unlike the monomeric state of ABA receptor subfamilies I and II (RCAR1–RCAR10), the members of subfamily III are dimeric receptors, except for RCAR13 that exists in a state of monomer–dimer shift (Nishimura *et al.*, 2009; Dupeux *et al.*, 2011; Hao *et al.*, 2011). The dimeric receptors inhibit the activity of PP2Cs in an ABA-dependent manner and possess less sensitivity to ABA than do the monomeric receptors (Ma *et al.*, 2009; Tischer *et al.*, 2017). Despite this lower sensitivity, genetic evidence suggests that the dimeric receptors are important in all ABA responses measured to date (Okamoto *et al.*, 2013; Ye *et al.*, 2017).

A recent study revealed that CARK1 interacts with and phosphorylates RCAR11 at the site of T78 (Zhang *et al.*, 2018). On the basis of the similarity of subfamily III receptors (Ma *et al.*, 2009; Park *et al.*, 2009), it is reasonable to hypothesize that an interaction might exist between the other members of subfamily III receptors and CARK1. In this study, we confirmed this hypothesis by GST pull-down, BiFC, and Co-IP assays (Fig. 1), suggesting that a kinase could target several receptors and regulate ABA signaling in plants.

The kinase assay *in vitro* revealed that recombinant CARK1 protein could phosphorylate RCAR12/13/14 (Fig. 2B–D), indicating a complex regulation of RCARs through phosphorylation. A recent study showed that the TOR kinase phosphorylates ABA receptors (RCAR12/PYL1S119 and RCAR10/PYL4S114) at a conserved serine residue to inhibit the binding of RCARs to ABA and then disrupts the ABA signaling pathway (Wang *et al.*, 2018). Moreover, Arabidopsis EL1-like (AEL) casein kinases phosphorylate RCARs and promote their ubiquitination and degradation, ultimately leading to suppression of the ABA response (Chen *et al.*, 2018). However, our lab previously found that CARK1 phosphorylates RCAR3 or RCAR11 to activate the ABA pathway (Zhang *et al.*, 2018). The phosphorylated RCARs could alter their structure, sheltering from ABA binding (Wang *et al.*, 2018), or exposing ubiquitination sites to be degraded by the 26S protease (Chen *et al.*, 2018). Here, we confirmed that the phosphorylation of RCAR11 by CARK1 contributed to ABA signaling and stress responses. Therefore, we could propose that the phosphorylation of RCARs might play different roles in ABA signaling.

RCARs are highly conserved in plants, and phosphorylation of RCARs by CARK1 confers the possibility to modulate ABA signaling. Our results showed that the interaction of CARK1 with RCAR12, RCAR13, or RCAR14 would activate the ABA signaling pathway by inducing the expression of the ABA-responsive genes. Interestingly, GST pull-down assay demonstrated that CARK1 could physically interact with RCARs *in vitro* (Supplementary Fig. S3), but not every ABA receptor was found to interact with CARK1 *in vivo* (Supplementary Fig. S1). This is due to the fact that the structures of protein in an *E. coli* system are not identical to those in Arabidopsis. In this study, we demonstrate that subfamily III receptors (RCAR12, RCAR13, and RCAR14) physically interact with CARK1 and are phosphorylated by CARK1 at T105, T101, and S81, respectively, indicating that phosphorylation of RCAR12, RCAR13, or RCAR14 positively regulates ABA signaling. The phosphor-defective form of RCAR12/13/14 proteins presented a weak phosphorylated signal, which may due to other possible phosphosites phosphorylated by CARK1. Therefore, we propose that CARK1 tends to interact with and phosphorylate subfamily III receptors, and T105 of RCAR12, T101 of RCAR13, and S81 of RCAR14 are the major phosphosites of CARK1.

Further analysis confirmed that the RCAR11^T78E^ (a phosphor-mimic form) transgenic plants in the 1124 background were more sensitive to ABA than the 1124 mutant plants. In contrast, the RCAR11^T78A^ (a phosphor-defective form) plants were less sensitive. Hence, the phosphorylation of RCAR11 could modulate ABA signaling. The complementation of *CARK1* in the 1124 mutant could rescue the sensitivity of plants to ABA, indicating that CARK1 phosphorylates ABA receptors and is involved in ABA signaling.

The analysis of ABA-mediated physiological responses revealed that *CARK1*:1124 transgenic plants were more sensitive to ABA than 1124 plants but less sensitive than *CARK1*-OE plants (Fig. 4). Therefore, CARK1 may phosphorylate other ABA receptors, such as RCAR13, which is consistent with the fact that CARK1 phosphorylates ABA receptors to modulate fine-tuning of ABA signaling. Interestingly, the R11:1124 transgenic plants were sensitive to ABA, being similar to R11E:1124 transgenic plants (Fig. 3), or even more sensitive (Fig. 3). We propose that one or more unknown kinases may also phosphorylate RCAR11 and then the ABA signal pathway is activated under stresses. In conclusion, the phosphorylation of ABA receptors plays an important role in ABA signal transduction, and CARK1 together with RCAR11/12/13/14 synergistically regulates ABA signaling, although the detailed mechanism is still unknown.

## Supplementary data

Supplementary data are available at *JXB* online.

Fig. S1 Controls of the BiFC assay.

Fig. S2 Construct used for plant and identification of transgenic lines by qRT-PCR and protein levels.

Fig. S3 *In vitro* GST pull-down assay of CARK1-KD interaction with RCARs.

Table S1. Primers used in this study.

## Supplementary Material

Supplementary Material
