# Supplementary material for: CARK1 phosphorylates subfamily III members of ABA receptors
Source: J Exp Bot. 2018 Oct 30;70(2):519–28. doi: 10.1093/jxb/ery374 (PMC6322571; doi:10.1093/jxb/ery374)
Supplement: Supplementary Material [file ery374_suppl_supplementary_material.pdf]

Supporting Information

Article title: **CARK1 phosphorylates ABA receptors subfamily III members**

Authors: Xiaoyi Li¶, Xiangge Kong¶, Qi Huang¶, Hu Ge, Qian Zhang, Liang Zhang, Gaoming Li , Lu Peng, Zhibin Liu, Jianmei Wang, Xufeng Li, Ying Yang*

*Author for correspondence: Yi Yang (yangyi528@scu.edu.cn)
¶ These authors contributed equally to this work

The following Supporting Information is available for this article:

Supporting Online Figures (Fig. S1—S2)

Supporting Online Table (Table S1)

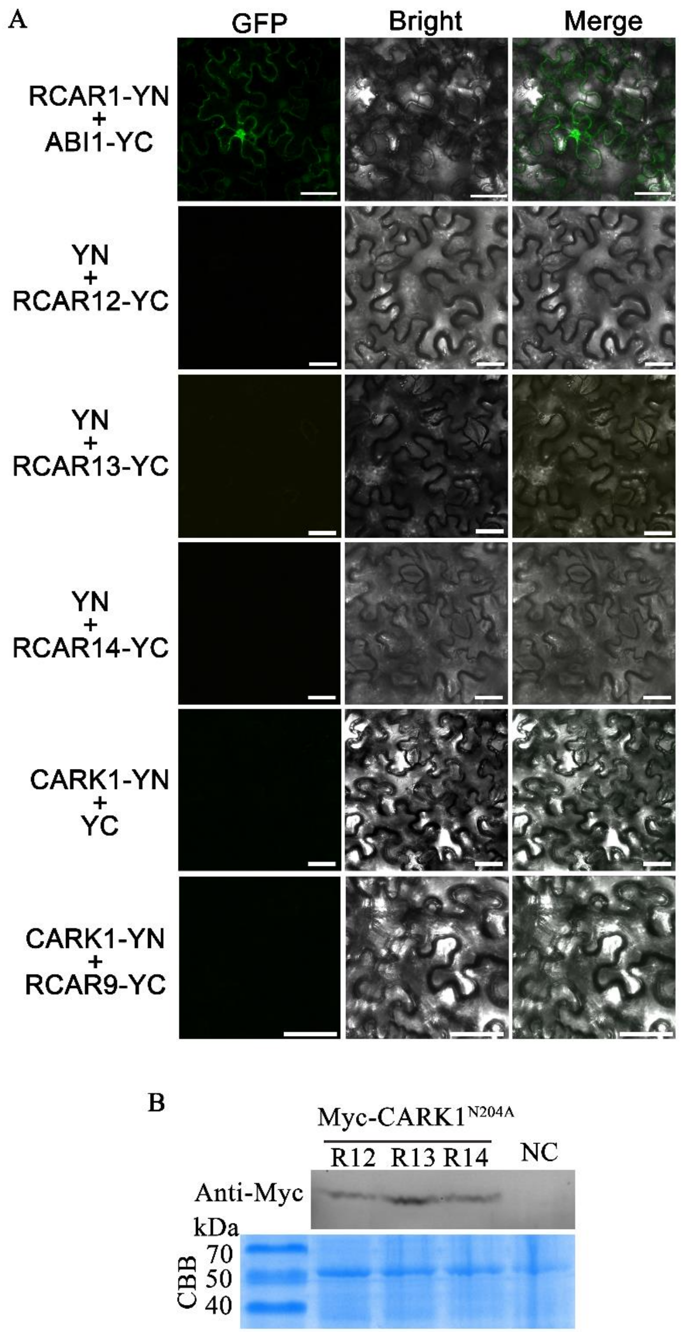

Supplementary Fig. S1 Controls of BiFC assay. A, Co-expression of RCAR1-YN and ABI1-YC (positive control) in leaves of *N. benthamiana*, yielded fluorescence signals both in the cytosol and in the nucleus. No signal of YFP fluorescence was detected after co-expression of YN and with RCAR12-YC, RCAR13-YC，RCAR14-YC, YC and RCAR9-YC with CARK1-YN. Scale bar, 50 μm. **B,** Western blot analyzing CARK1^{N204A} expression levels in tobacco epidermal cells, co-infiltration CARK1^{N204A} with RCAR12, RCAR13 and RCAR14, respectively. NC, Crude extracts without plasmids transfection were used as the negative control.

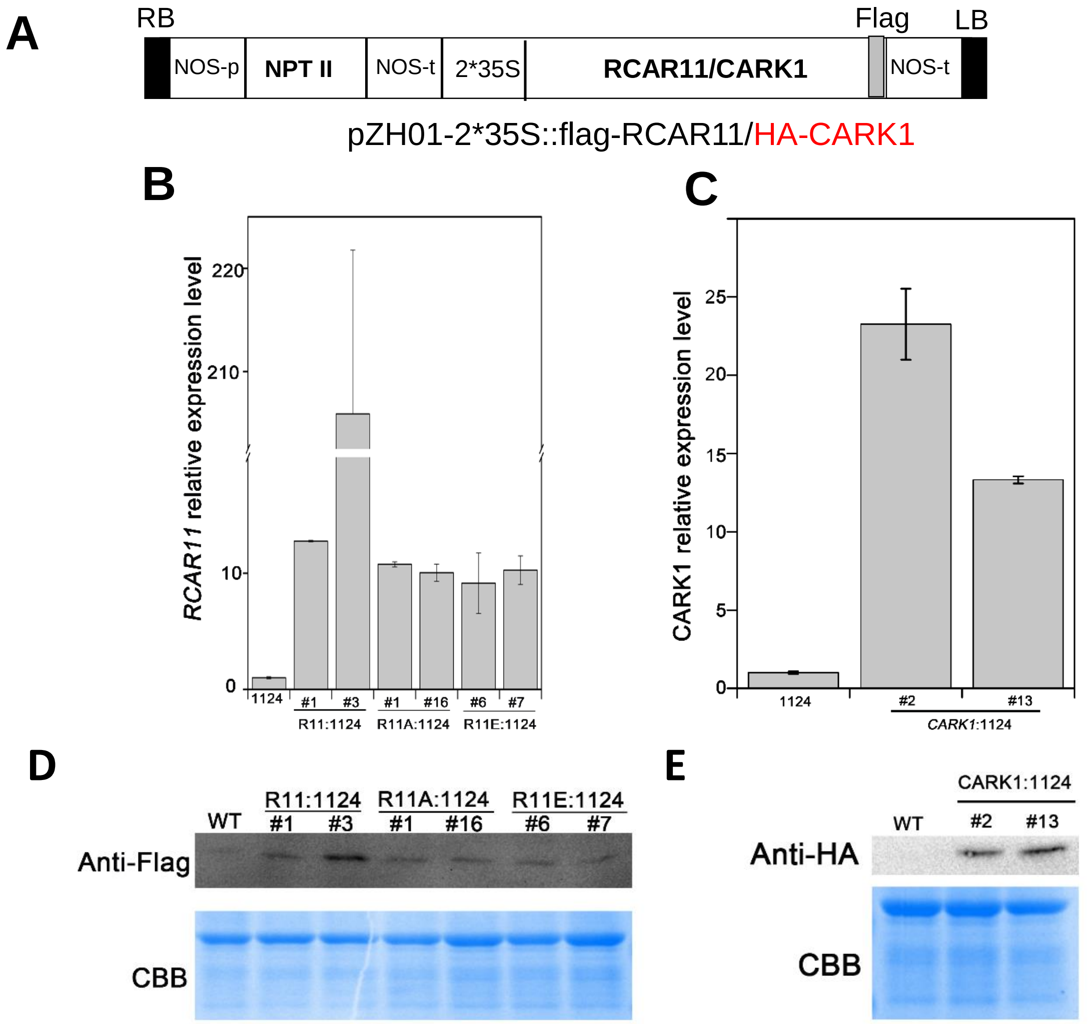

Supplementary Fig. S2 Construct used for plant and identification of transgenic lines by qRT-PCR and protein levels. **A,** Construct used for transgenic plant, RB, right T-DNA border, LB, left T-DNA border; Nos-p, NOS promoter; NOS-t, NOS terminator, 35S, cauliflower mosaic virus (CaMV) 35S promoter; NPTII, neomycin phophotransferase II. **B and C**, Real time-PCR analysis of the 1124, R11:1124, R11A:1124, R11E:1124, *CARK1*:1124. Expression levels of *RCAR11* or *CARK1* transcripts were determined by qRT-PCR using gene-specific primers. ACTIN2/8 mRNA level were used as the internal control. **D and E**, western blot analyzing protein levels of transgenic plants. Anti-Flag was used to detect RCAR11:1124, RCAR11A:1124 and RCAR11E:1124, and anti-HA was used to detect CARK1:1124. CBB (Coomassie Brilliant Blue) stain is shown as loading control.

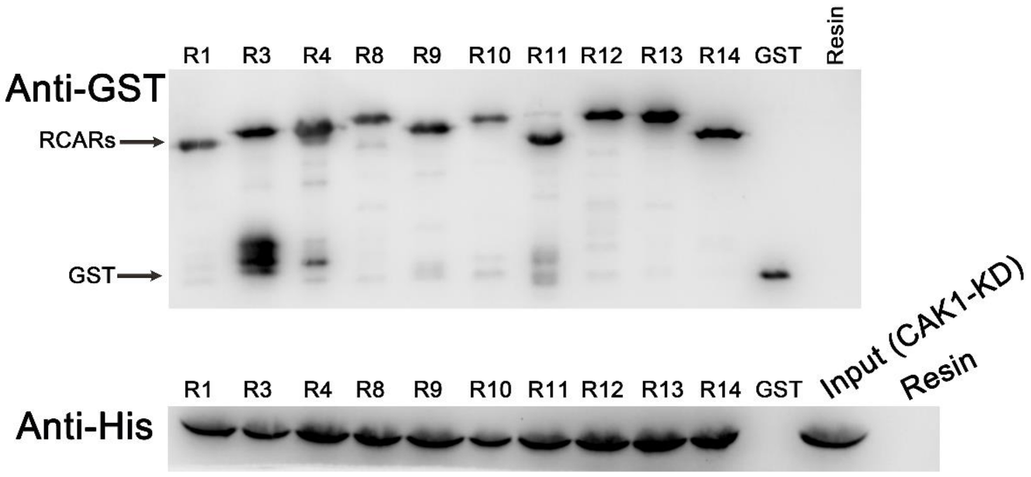

Supplementary Fig. S3 ***In vitro*** **GST pull-down assay of CARK1-KD interaction with RCARs.**

6xHis fused CARK1-KD was incubated with, GST-tagged RCARs as indicated, respectively. The RCARs proteins, GST or the negative control (Resin) were analyzed by anti-GST (up) and immunoblotting with antibodies against the His-tag (bottom).

Table S1 Primers used in this paper

Assays	Genes	Primer sequences (5`-3`)
Primers for GST-pull down	RCAR1	F (*EcoR*I): GGAATTCATGGACGGCGTTGAAGGCG
		R (*Xho*I): CCGCTCGAGTCACTGAGTAATGTCCTGAGAAGCC
	RCAR3	F (*EcoR*I): GGAATTCATGGAAGCTAACGGGATTG
		R (*Xho*I): CCGCTCGAGTCAAGCGTAATCTGGAACATC
	RCAR4	F (*EcoR*I): GGAATTCATGAACGGTGACGAAACAAAGAAG
		R (*Xho*I): CCGCTCGAGTATCTTCTTCTCCATAGATTCT
	RCAR8	F (*BamH*I): GGAATTCATGGAAGCTAACGGGATTG
		R (*EcoR*I): CCGCTCGAGTCAAGCGTAATCTGGAACATC
	RCAR9	F (*EcoR*I): GGAATTCATGCCAACGTCGATACAGTT
		R (*Xho*I): CCGCTCGAGTTACGAGAATTTAGAAGTGTTCTCG
	RCAR10	F (*EcoR*I): GGAATTCATGCCAACGTCGATACAGTT
		R (*Xho*I): CCGCTCGAGCAGAGACATCTTCTTCTTGCTC
	RCAR11	F (*BamH*I): CGCGGATCCATGGCGAATTCAGAGTCCTC
		R (*Sma*I): TCCCCCGGGCCTAACCTGAGAAGAGTTGT
	RCAR12	F (*BamH*I): CGCGGATCCATGGCGAATTCAGAGTCCTC
		R (*Sma*I): TCCCCCGGGCCTAACCTGAGAAGAGTTGT
	RCAR13	F (*EcoR*I): GGAATTCATGAATCTTGCTCCAATCCA
		R (*Xho*I): CCGCTCGAGTCAGGTCGGAGAAGCCGTGG
	RCAR14	F (*EcoR*I): GGAATTCATGAGCTCATCCCCGG
		R (*Xho*I): CCGCTCGAGTTATTCATCATCATGCATAGGTG
Primers for BiFC	RCAR9	F (*BamH*I): CGGGATCCATGCCAACGTCGATACAGTTT
		R (*Sal*I): TCCGTCGACCGAGAATTTAGAAGTGTTCTCG
	RCAR12	F (*BamH*I): CGCGGATCCATGGCGAATTCAGAGTCCTC
		R (*Sal*I): TCCGTCGACAACCTGAGAAGAGTTGT
	RCAR13	F (*BamH*I): CGCGGATCCATGAATCTTGCTCCAATCCA
		R (*Sal*I): TCCGTCGACGGTCGGAGAAGCCGTGG
	RCAR14	F (*BamH*I): CGCGGATCCATGAGCTCATCCCCGG
		R (*Sal*I): TCCGTCGACTTCATCATCATGCATAGGTG
Primers for CoIP	RCAR12	F (*BamH*I): CGCGGATCCATGGCGAATTCAGAGTCCTC
		R (*Sal*I): TCCGTCGACAACCTGAGAAGAGTTGT
	RCAR13	F (*BamH*I): CGCGGATCCATGAATCTTGCTCCAATCCA
		R (*Sal*I): TCCGTCGACGGTCGGAGAAGCCGTGG
	CARK1/ CARK1m	F (*Sal*I): TCCGTCGACATGGGCTGCTTTGGTTGTTGT
		R (*Xba*I): GCTCTAGAATACGGGTTCCTGTGTGGAGTC
Primers for site-directed mutagenesis	RCAR12^{T105A}	CGAGTGGGATGCGCGCGCGACGTGAAC
		CGCGCATCCCACTCGCATCTCGAAATC
	RCAR13^{T101A}	GATTAAAGTTGGGGCGATAAGGGAAG
		CGCCCCAACTTTAATCTCTTTGATGC
	RCAR14^{S81A}	GGTGACGTCGGAGCGGTCAGAGAAG
		CGCTCCGACGTCACCATCACCGGAG
